# Deep haplotype analyses of target‐site resistance locus *ACCase* in blackgrass enabled by pool‐based amplicon sequencing

**DOI:** 10.1111/pbi.14033

**Published:** 2023-04-10

**Authors:** Sonja Kersten, Fernando A. Rabanal, Johannes Herrmann, Martin Hess, Zev N. Kronenberg, Karl Schmid, Detlef Weigel

**Affiliations:** ^1^ Institute of Plant Breeding, Seed Science and Population Genetics University of Hohenheim Stuttgart Germany; ^2^ Department of Molecular Biology Max Planck Institute for Biology Tübingen Tübingen Germany; ^3^ Agris42 GmbH Stuttgart Germany; ^4^ Pacific Biosciences Menlo Park California USA

**Keywords:** Amplicon sequencing, HiFi long reads, *pbaa*, *Alopecurus myosuroides*, herbicide resistance, *ACCase*

## Abstract

Rapid adaptation of weeds to herbicide applications in agriculture through resistance development is a widespread phenomenon. In particular, the grass *Alopecurus myosuroides* is an extremely problematic weed in cereal crops with the potential to manifest resistance in only a few generations. Target‐site resistances (TSRs), with their strong phenotypic response, play an important role in this rapid adaptive response. Recently, using PacBio's long‐read amplicon sequencing technology in hundreds of individuals, we were able to decipher the genomic context in which TSR mutations occur. However, sequencing individual amplicons are costly and time‐consuming, thus impractical to implement for other resistance loci or applications. Alternatively, pool‐based approaches overcome these limitations and provide reliable allele frequencies, although at the expense of not preserving haplotype information. In this proof‐of‐concept study, we sequenced with PacBio High Fidelity (HiFi) reads long‐range amplicons (13.2 kb), encompassing the entire *ACCase* gene in pools of over 100 individuals, and resolved them into haplotypes using the clustering algorithm PacBio amplicon analysis (*pbaa*), a new application for pools in plants and other organisms. From these amplicon pools, we were able to recover most haplotypes from previously sequenced individuals of the same population. In addition, we analysed new pools from a Germany‐wide collection of *A. myosuroides* populations and found that TSR mutations originating from soft sweeps of independent origin were common. Forward‐in‐time simulations indicate that TSR haplotypes will persist for decades even at relatively low frequencies and without selection, highlighting the importance of accurate measurement of TSR haplotype prevalence for weed management.

## Introduction

Since the introduction of herbicides in agriculture in the 1940s, numerous plant species have evolved resistance to these chemicals. The two main mechanisms that led to rapid adaptation are non‐target‐site resistance (NTSR) and target‐site resistance (TSR). NTSR refers to processes that degrade or physically prevent the active ingredient from reaching its target, such as enhanced metabolization, decreased absorption or translocation and sequestration (Devine and Shukla, 2000; Heap, 2014b). NTSR typically involves multiple genes (Cai *et al*., 2022; Franco‐Ortega *et al*., 2021; Kreiner *et al*., 2021; Van Etten *et al*., 2020), resistance is often quantitative and several candidate gene families contribute to it, including cytochromes P450 monooxygenases, glycosyltransferases or glutathione S‐transferases (reviewed in Gaines *et al*., 2020). TSR has more qualitative effects, it is usually characterized by resistance to high levels of the herbicide, and it can often be traced back to large‐effect gene mutations that change individual amino acids in herbicide target enzymes. More rarely, TSR is associated with overexpression of the target enzyme (Devine and Shukla, 2000).

The first TSR mutation was discovered in the *psbA* gene (Golden and Haselkorn, 1985). The *psbA* product, chlorophyll‐binding protein D1, normally binds plastoquinone and serves as an essential component of photosystem II (PS II). The herbicide triazine competes with plastoquinone at the plastoquinone‐binding site of protein D1, thus inhibiting PS II electron transport (reviewed in Gronwald (1997)). The amino acid substitution Ser‐264‐Gly prevents triazine binding, while still allowing plastoquinone binding (Gronwald, 1997). However, it comes with deleterious effects on CO_2_ assimilation and plant development (Ireland *et al*., 1988; Ort *et al*., 1983). In the following decades, TSR mutations were identified in other genes including the genes for L‐tubulin (Anthony *et al*., 1998; Chu *et al*., 2018; Délye et al., 2004a; Hashim *et al*., 2012; Yamamoto *et al*., 1998), acetolactate synthase (*ALS*) (Délye and Boucansaud, 2007; Tranel and Wright, 2002), acetyl‐CoA carboxylase (*ACCase*) (reviewed in Kaundun (2014)) and 5‐enolpyruvylshikimate‐3‐phosphate synthase (*EPSPS*) (reviewed in Sammons and Gaines (2014)). In some cases, only a single amino acid substitution has been found to confer herbicide resistance, while in other genes, including *ALS* and *ACCase*, mutations at several residues can lead to herbicide resistance.

Many weeds have evolved independent resistances to multiple herbicides. Among them are European populations of the grassy weed *Alopecurus myosuroides*, where herbicide resistance results in significant yield losses for farmers (Rosenhauer *et al*., 2013; Varah *et al*., 2019). In fact, widespread resistance to ACCase inhibitors in *A. myosuroides* has greatly limited the ability of farmers to effectively control this problematic weed (Délye *et al*., 2010; Heap, 2014a; Hess *et al.*, 2022; Rosenhauer *et al*., 2013). Aryloxyphenoxy‐propionates (FOPs), phenylpyrazolines (DENs) and cyclohexanediones (DIMs) all block the first step in fatty acid synthesis by inhibiting ACCase catalytic activity (Walker *et al*., 1988). These herbicides act specifically on grasses because they target the homomeric plastidic ACCase, which is almost exclusively found in monocots and which is encoded in the nuclear genome (Incledon and Hall, 1997). All seven known sites at which TSR mutations occur are located in the penultimate exon, which encodes the C‐terminal domain: Ile1781, Trp1999, Trp2027, Ile2041, Asp2078, Cys2088 and Gly2096. Depending on the mutation, amino acid substitutions confer resistance to one or several of the three different classes of ACCase inhibitor herbicides, Ile1781Leu and Asp2078Gly being resistant to all three classes (Beckie and Tardif, 2012). In *A. myosuroides*, plants with the Trp2027Cys and Ile2041Asn mutations survive treatments with FOPs and DENs, while Gly2096 confers resistance exclusively to FOPs (Délye, 2005; Délye *et al*., 2008; Petit *et al*., 2010). The degree of cross‐resistance provided by TSRs is thought to be one of the factors that determine the frequency at which they are found (Gaines *et al*., 2020; Powles and Yu, 2010). Another important factor comes from the effect each mutation has on herbicide‐independent plant fitness. For instance, mutations at the most frequently affected site, Ile1781, appear to have no deleterious fitness effect (Délye *et al*., 2013b; Menchari *et al*., 2008). On the other hand, plants carrying the Asp2078Gly allele are shorter, have less vegetative dry biomass and set fewer seeds. Similarly, plants with the Trp2027Cys allele have lower seed production (Du *et al*., 2019; Menchari *et al*., 2008; Vila‐Aiub *et al*., 2015). The frequencies of *ACCase* TSR mutations have been investigated in several studies (Délye *et al*., 2010; Délye *et al*., 2004b; Menchari *et al*., 2006; Rosenhauer *et al*., 2013), but usually without considering the genomic context of the complete *ACCase* gene. Complete haplotype information is important in several respects, including establishing the number of times with which a specific mutation has occurred independently, and whether TSR mutations occur preferentially on specific haplotype backgrounds (Kersten *et al*., 2023; Kreiner *et al*., 2022).

Pool sequencing with Illumina short reads offers a cost‐ and time‐saving option for the analysis of many individuals by combining barcoded DNA from multiple samples before sequencing (Ferretti *et al*., 2013; Schlötterer *et al*., 2014). This has included pooled amplicon sequencing approaches for resistance diagnosis assays in multiple species (Délye *et al*., 2020, 2015; Schlipalius *et al*., 2019). Unfortunately, due to the limited read lengths (from 50 to 300 bases in paired‐end mode), to preserve haplotype information of an entire gene, variant calls have to be phased based on known patterns of linkage disequilibrium, with phasing accuracy depending on the co‐occurrence of variants within paired‐end reads. Long‐read amplicons offer many advantages to solve the above‐mentioned limitations. The most widely used third‐generation long‐read sequencing technologies are from Pacific Biosciences (PacBio) and Oxford Nanopore Technologies (ONT). Unfortunately, in their native form, both suffer from limited per‐base accuracy (below 90%), which until recently made reliable variant calling or haplotype determination in ONT and PacBio long reads difficult (Korlach, 2013). With the introduction of the PacBio circular consensus sequencing strategy to generate High Fidelity (HiFi) reads (Wenger *et al*., 2019), random sequencing errors can now be corrected, and average per‐base accuracy above 99% (q20) can now be routinely achieved (Travers *et al*., 2010; Wenger *et al*., 2019). In combination with the new clustering software PacBio amplicon analysis (*pbaa*) from Pacbio (Kronenberg *et al*., 2021), this offers completely new perspectives for the application of amplicon sequencing in diagnostics.

In this study, we describe a high‐throughput PacBio amplicon workflow for pooled samples that can be easily adapted to any gene of interest, independently of the organism. We demonstrate its feasibility with the TSR gene *ACCase*, for which we amplified a ~14 kb long fragment, which includes 585 bp upstream of the CDS, the 32 exons and 31 introns of the gene (12.5 kb) and 364 bp downstream. We provide a detailed hands‐on laboratory protocol to amplify and long‐read sequence loci such as *ACCase* as well as analysis recommendations for using the software *pbaa* in pools with up to 200 samples. We applied this workflow to German field populations of *A. myosuroides*. With the exception of a few low‐frequency haplotypes, we were able to recover all individual haplotypes in the pools we tested. Furthermore, we found TSRs resulting from soft sweeps in almost all populations. Using SLiM simulations, we demonstrate that these TSR mutations may persist in field populations for decades to centuries, depending on their starting allele frequencies, even when selection is no longer applied. Therefore, it is strongly advised not only to base weed management strategies solely on herbicide applications but also to integrate mechanical weed management and crop rotation, to keep the incidence of weeds in the field continuously low with a combination of chemical and non‐chemical measures.

## Results

### Workflow to sequence and analyse long‐read amplicon pools

In a recent study, we sequenced PacBio long‐read amplicons of the TSR locus *ACCase* in individuals of 47 European *A. myosuroides* populations (Kersten *et al*., 2023). We discovered a recurrent pattern within field populations of different haplotypes with the same TSR mutation resulting from independent mutation events, as opposed to the same TSR mutation being transferred to other haplotypes by recombination. Characterizing the TSR diversity of entire haplotypes to this level of resolution was enabled by two main factors: sequencing of single individuals with HiFi reads and the clustering of these reads to reconstruct both haplotypes in each individual with the *pbaa* tool (Kronenberg *et al*., 2021). However, performing independent DNA extractions and generating long‐range amplicons with dual barcodes per individual proved to be both time‐consuming and costly. To mitigate these limitations in future studies, we evaluated whether haplotype‐level resolution can be achieved by sequencing per‐field pools of large numbers of individuals. This is of interest for the further characterization of the origin and evolutionary tempo of herbicide resistance.

**Figure 1:**
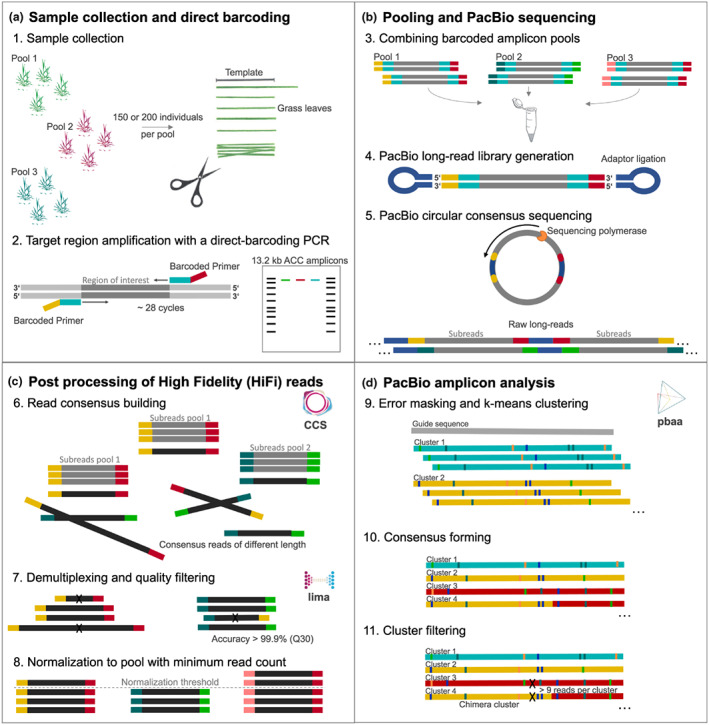
Workflow to generate and analyse long‐read amplicons in pools. (a) Leaf material is collected using a size template to ensure equal sample representation in each pool. Long‐range amplicon products are obtained by PCR with direct barcoding to individually tag each pool. Products are visualized by gel electrophoresis for quality control and validation of amplicon concentration measurements. (b) All population pools are combined in equal amounts in a single tube. A PacBio library is generated and sequenced in circular consensus mode on a Sequel II system. (c) Computational processing includes read‐consensus building, demultiplexing and filtering of raw reads. (d) *pbaa* clustering is used for variant detection and filtering (Kronenberg *et al.*, 2021). The output is *fasta* files listing all haplotypes per population pool and including meta information on read coverage of each haplotype.

For benchmarking purposes, we selected nine populations from our previous study (Kersten *et al*., 2023) and compared the *ACCase* haplotypes determined from 22 to 24 independently sequenced individuals to *ACCase* haplotypes inferred from pools of 200 individuals. Each population was sown separately in the greenhouse. Then, we used a paper‐size template to harvest similar amounts of 4‐week‐old leaf tissue from each plant and pooled them per population prior to DNA extraction (Figure 1a). Next, from 50 ng of DNA (on average ~65 diploid genome copies per individual in the pool; see Experimental procedures), we amplified a 13.2 kb long‐range PCR fragment that encompasses the entire *ACCase* coding sequence including introns, plus 585 bp upstream and 364 bp downstream sequences. We used direct dual‐indexing per pool, which later allowed multiplexing of all pools on a single SMRT cell. We paid special attention to combine similar amounts of PCR amplicons from all pools, by determining amplicon concentrations with a Qubit fluorometer and an additional gel electrophoresis for cross‐validation before combining the pools (Figure 1a). A PacBio amplicon library was then created, size‐selected using a BluePippin system and sequenced on the Sequel II system (Figures 1b, S1).

The current practice for *de novo* assembly studies, structural variant calling and amplicon analyses is to start from q20 HiFi reads, that is reads with an accuracy of at least 99% (Travers *et al*., 2010; Wenger *et al*., 2019). Since we were working with pools consisting of hundreds of individuals, our downstream analysis relied heavily on the precision of each individual read. Therefore, we increased the quality of the input HiFi reads to q30 (≥99.9% accuracy; Figure 1c). To compare haplotype frequencies between populations, we normalized all HiFi reads to the pool with the lowest number of reads, 16000 reads, corresponding to an average read depth of 40 for each amplicon represented in the sample (200 diploid individuals). Since the most common error types of HiFi reads are indels in homopolymer contexts (Travers *et al*., 2010; Wenger *et al*., 2019), we applied further filters including ‘minimum cluster‐read‐count 20’ (half the expected depth per single haplotype) and ‘minimum‐cluster‐frequency 0.00125’, which referred to the fraction of reads to support a true cluster in our data sets (Figure 1d).

### Individuals versus pools – a *pbaa* cluster quality assessment

**Figure 2:**
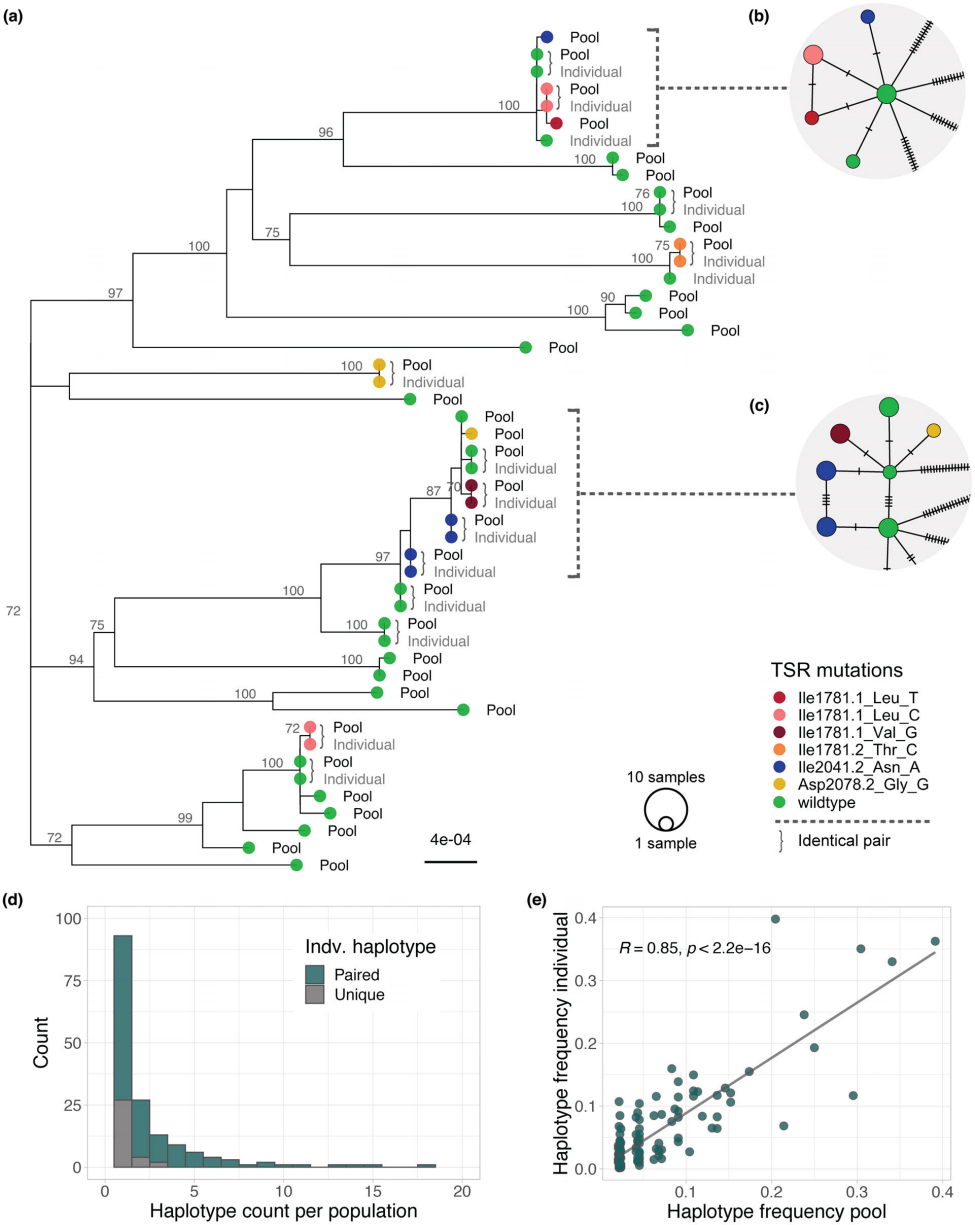
Unique *ACCase* haplotypes identified by *pbaa* in individuals compared to pools for the same population. (a) Maximum‐likelihood tree of haplotypes identified in the pool data set (200 samples), and haplotypes inferred in the individual data set (24 samples). Samples were collected in an agricultural field in Belgium (BE01585). For simplicity, in the individual data set, only unique haplotypes are shown. Tree labels indicate the data set of origin (Pool vs. Individual). Coloured tree tips show target‐site‐resistance (TSR) mutations. Curly brackets mark identical haplotype pairs found in both the individual and the pool data set from the same population. (b, c) Haplotype network representing the corresponding clade in the tree. *pbaa* can successfully recover haplotypes that differ only in one mutation (tick bar). (d) Haplotype counts per population in the individual data set. The number of haplotypes that could have been successfully identified in the pool data set is marked in green. Only a fraction of the low abundant ones could not be recovered (grey). (e) Correlation of haplotype frequencies in the pool data set versus the individual data set.

*pbaa* has been exclusively tested either on single individuals of diploid or polyploid species or on up to six HLA genes of the same individual (Kronenberg *et al*., 2021). After read‐to‐read alignment, for each focal read, *pbaa* sorts the alignments in decreasing identity and retains only the top ‘n’ alignments, which we call a pile. The frequency of each haplotype in the pool affects the parameter choices for *pbaa*. For a perfectly balanced pool, where every haplotype has the same number of reads, the pile size should match the expected haplotype read count. Therefore, to reduce spurious cluster formation, we adjusted the pile size to be about a quarter larger than the expected haplotype read count. The pile is used for error correcting each focal read. If the pile size is set too high, the pile will contain many cross‐haplotype alignments and the haplotype‐specific variant in the focal read will be corrected away. Similarly, the minimum variant frequency within a pile can affect which variants are masked out. Assuming the pile contains a high fraction of within haplotype alignments, a variant frequency cut‐off of 0.4 performs well across a range of parameters. We then compared the resulting haplotypes in pools to haplotypes inferred from individuals of the same populations (Figure 2, Table 1).

**Table 1:** Individual haplotype recovery in pools

Population	Number of haplotypes: pool	Number of haplotypes: individuals	Number of correct pairs	Number of correct TSR pairs	Unidentified TSRs in pools	Additional TSRs in pools
BE01585	34	15	13 (15)	7 (7)	0	3
DE01467	26	16	14 (16)	2 (2)	0	2
DE01580	31	18	14 (18)	3 (3)	0	3
FR01434	41	21	17 (21)	3 (4)	1	7
FR01729	35	24	19 (24)	3 (4)	1	6
FR03200	24	11	8 (11)	3 (3)	0	3
FR07250	39	18	14 (18)	7 (10)	3	12
NL01505	26	22	19 (22)	2 (2)	0	0
UK06481	34	19	13 (19)	4 (5)	1	6

The Belgium population BE01585 had a high diversity of TSR haplotypes of independent origin, a classical sign of soft sweeps due to herbicide selection pressure. Among the original 24 diploid individuals, we identified a total of 15 unique haplotypes, seven of which are haplotypes with TSR mutations (Table 1). In the pool data set, we successfully recovered 13 of the original 15 unique haplotypes identified in individuals, including all TSR haplotypes (Figure 2a). The two missing haplotypes in the pool were haplotypes found only in a single individual. Moreover, since the pool contained a larger number of individuals, we could identify 19 additional rare haplotypes, three of which were also TSR haplotypes. Notably, *pbaa* applied to pools was able to correctly resolve haplotypes that differed by a single mutation (Figure 2b,c).

The ability to recover a haplotype in the pools was influenced by its prevalence in the population. All haplotypes that were present in at least four out of 24 individuals were found in the corresponding pool, as were more than 85% of haplotypes present in two or three individuals (Figure 2d). Haplotypes found in only one out of 24 individuals were recovered in 71% of cases. This is most likely a reflection of the experimental design, in which the pools and 24 individuals were drawn from the same seed lots, which contained thousands of seeds, but the 24 individuals were not a subset of the pools of hundreds of individuals. Nevertheless, there was a high correlation (*R* = 0.85, *P* < 2.2 e‐16) between haplotype frequency in individuals and in pools (Figure 2e). *pbaa* missed a few TSR haplotypes in the pools compared to the 24 individuals, but in all but one case, the analysis recovered additional TSR haplotypes in the pools. As one would expect from the deeper sampling, the number of haplotypes detected in the pools always exceeded the number of haplotypes found among the 24 individuals, from 15% to over twofold (Table 1). Thus, not only did the pools provide valuable, detailed information on the haplotype composition of field populations, but with the identification of up to 12 additional TSR haplotypes, they provided information of importance for resistance monitoring and herbicide use management (Hawkins *et al*., 2018; Powles and Yu, 2010). In addition, the collection of plant pools can constitute a valuable resource for the implementation of standardized epidemiological diagnostic methods, essential for monitoring future resistances (Comont and Neve, 2021).

### Haplotype clustering reveals the evolutionary context

We employed our pool approach to survey TSR haplotype diversity in a German‐wide contemporary collection of agricultural fields, for which seeds had been harvested in the year 2019. We selected 64 *A. myosuroides* populations collected in fields of winter annual crops: 49 populations that showed widespread resistance to the *ACCase*‐inhibiting herbicides Axial*®* (active ingredients 50 g/L of pinoxaden and 12.5 g/L cloquintocet‐mexyl), 13 populations with an incidence of Axial*®* resistance below 10% and two organic fields without a recent history of herbicide application (Data S1). Seventeen farms were represented with multiple fields. We used the same workflow as described above (Figure 1), but using pools of 150 individuals. To make results comparable across populations, the resulting HiFi reads were normalized to 5300 reads per pool, corresponding to an average read depth of 17.6 per chromosome (150 diploid individuals). The reads were filtered for ‘minimum cluster‐read‐count 9’ and ‘minimum‐cluster‐frequency 0.0017’, which led to an average number of 25 clusters per population (range 15–35).

**Figure 3:**
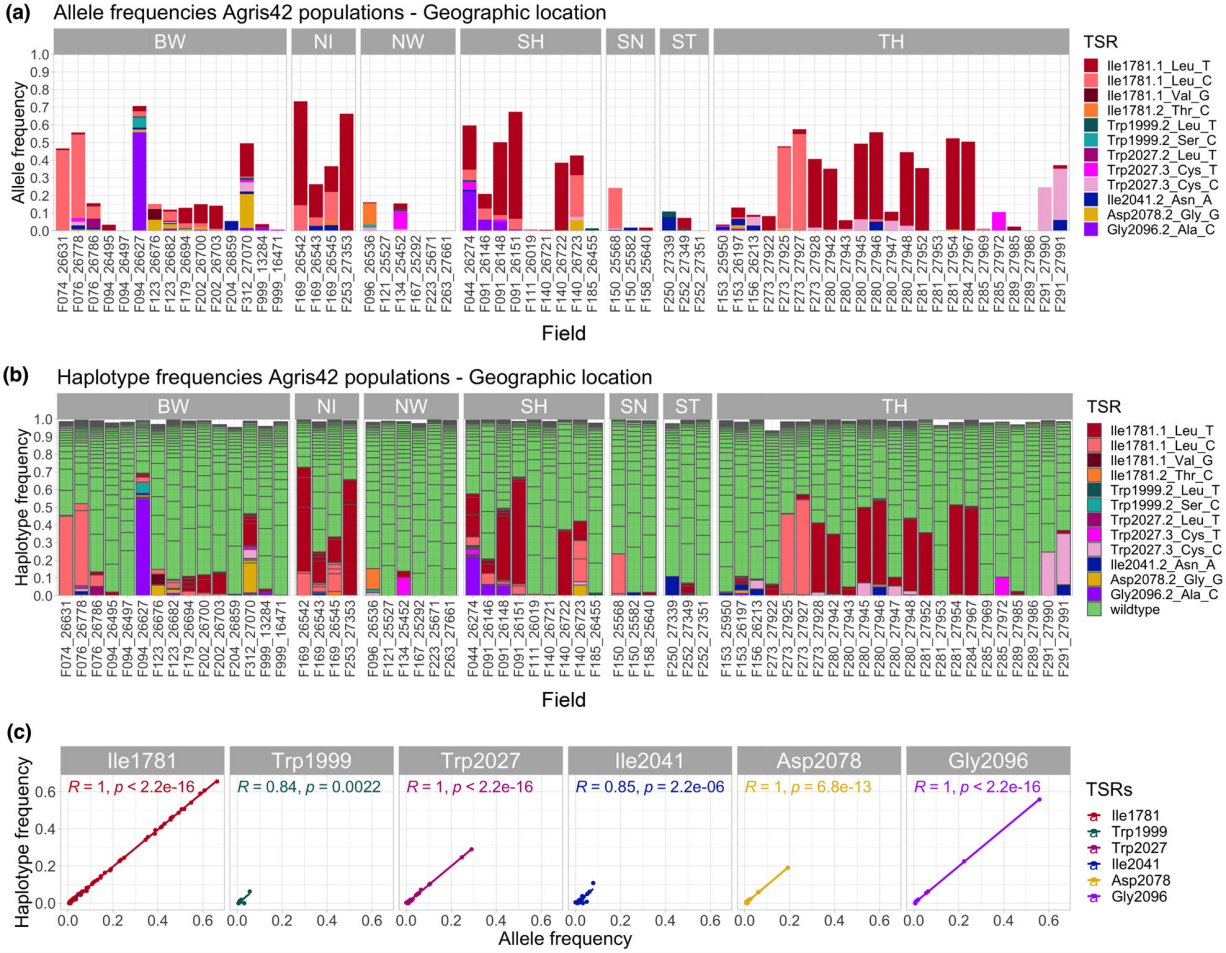
Comparison between conventional single‐nucleotide polymorphism (SNP) mapping and *pbaa* haplotype clustering. (a) TSR allele frequencies obtained by SNP mapping. Colours indicate different TSR mutations. (b) Haplotype frequencies were inferred using *pbaa* (Kronenberg *et al*., 2021). Colours refer to TSR and wild‐type haplotypes. (c) Correlation between allele frequencies and haplotype frequencies summarized per TSR amino acid position. Correlation coefficients and *P* values are shown separately in each TSR panel. BW, Baden‐Württemberg; NI, Lower Saxony; NW, North Rhine‐Westphalia; SH, Schleswig‐Holstein; SN, Saxony; ST, Saxony‐Anhalt; TH, Thuringia.

Conventional single‐nucleotide polymorphism (SNP) calling approaches have typically been used for variant calling and analysis of pooled data (Schlötterer *et al*., 2014), but they generally ignore the underlying genomic context. Based solely on allele frequencies, we can estimate the abundance of each TSR mutation (Figure 3a), but we do not know in which context they emerged (Figure 3b). To further assess the accuracy of the pooled clustering approach, we compared the TSR haplotype frequencies with allele frequencies from a conventional SNP calling approach (Figure 3a,b). Pearson correlation coefficients were highly significant and ranged from 0.85 to 1 for all six TSR mutations, with only a few low‐frequency *pbaa* clusters not captured (Figures 3c, S2).

The overall most common TSR mutation is Ile1781Leu, which has been reported in previous studies to increase the fitness of individuals in the absence of herbicide selection and therefore may have already existed in favourable conditions prior to herbicide application (Délye *et al*., 2013b; Du *et al*., 2019; Wang *et al*., 2010). While the number of populations per state was too small to make definitive statements about regional variation, the state with the smallest fields and farms, Baden‐Württemberg, had the most diverse set of TSR mutations and haplotypes. However, states also vary in their history of herbicide use and thus are not that easily comparable. Very few TSR mutations were observed in field populations of North Rhine‐Westphalia, Saxony and Saxony‐Anhalt, whereas the field populations in Thuringia seemed to be mainly dominated by single TSR haplotypes.

We refer to a hard sweep when a single haplotype dominates in a population. If, on the other hand, multiple adaptive haplotypes in a population increase in frequency at the same time, this is called a soft sweep (Hermisson and Pennings, 2017). In 38 of 55 German *A. myosuroides* populations containing TSR mutations, we can observe the latter phenomenon, confirming our previous results from European populations where herbicide adaptation occurred predominantly via soft sweeps through TSR mutations of independent origin (Kersten *et al*., 2023). We also find a significant proportion of NTSR for the *ACCase*‐inhibiting herbicide Axial*®* in this German data set, as the biotests reveal significantly more resistance than the TSR frequencies can explain (Figure S3a,c). However, the phenotypic resistance to Focus Ultra correlates significantly with the frequency of TSR mutations Ile1781Leu and Asp2078Gly, as reported before (Powles and Yu, 2010) (Figure S3b,d).

### Organically farmed fields show TSRs of independent origin

**Figure 4:**
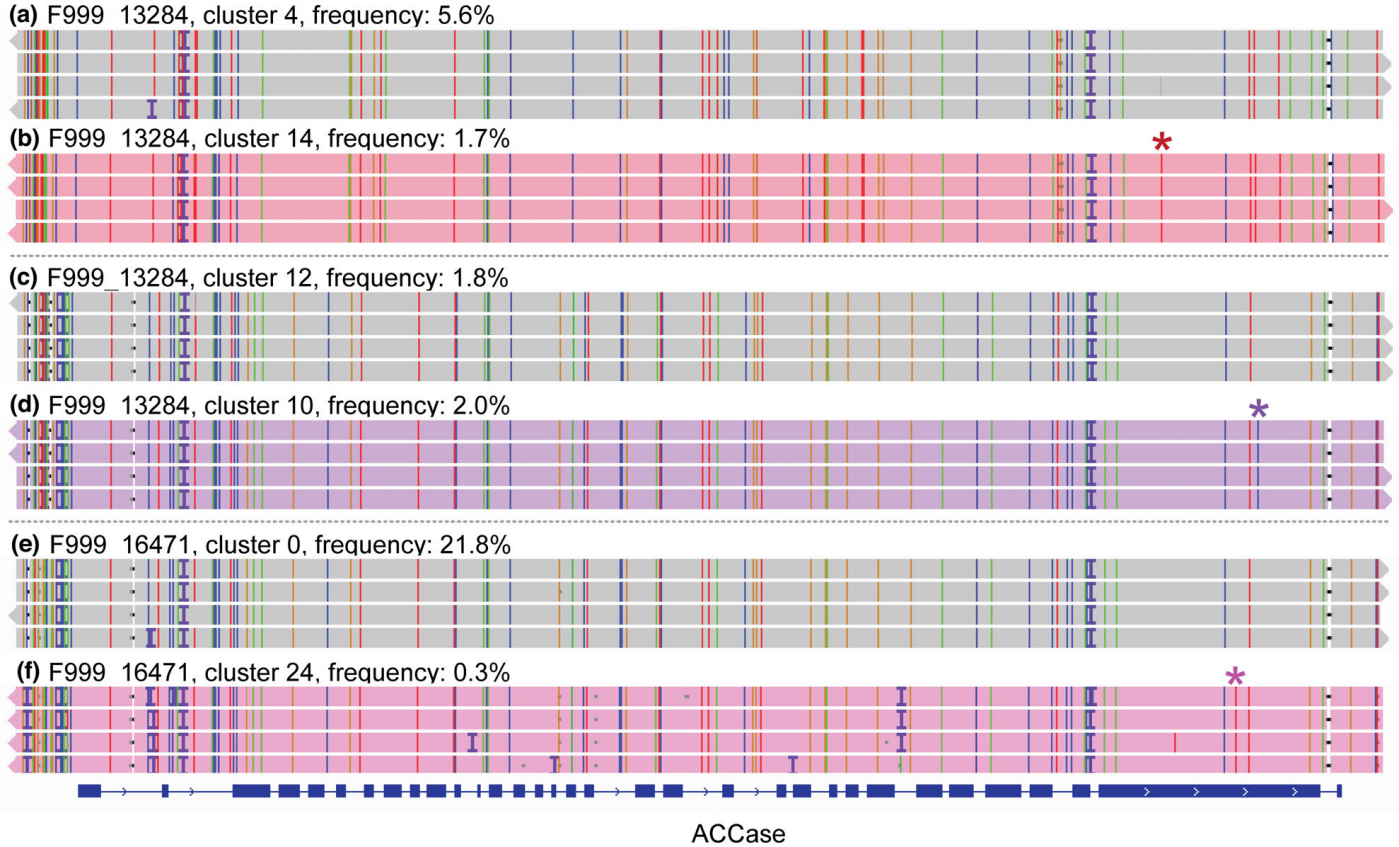
TSR haplotypes and the corresponding wild‐type haplotypes from which they arose in organic fields. (a, c, e) Wild‐type haplotypes, (b) a haplotype with the TSR mutation Ile1781.1Leu_T, (d) a haplotype with the TSR mutation Gly2096.2Ala_C, (f) a haplotype with the TSR mutation Trp2027.3Cys_T.

Among the nine phenotypically sensitive populations included in the study, there were two organically farmed fields that have not been treated with herbicides going back as far as at least 1980, which predates the introduction of *ACCase* inhibitors to the market. In these fields, we found TSR haplotypes at low frequencies, from 0.3% to 2.0% (Figure 4), in agreement with our previous inferences that standing genetic variation is the most likely evolutionary mechanism behind herbicide selection (Kersten *et al*., 2023). This is considerably higher than in a phenotyping‐based study of the grass *Lolium rigidum*, the frequency of resistant individuals to ALS inhibitors in untreated populations ranged from 0.001% to 0.012% (Preston and Powles, 2002). The observation of TSR mutations in organic fields without a history of herbicide use is in agreement with the *ACCase* TSR mutation Ile1781Leu having been detected in one out of 685 (0.146%, or 0.073% at the haplotype level) *A. myosuroides* herbarium specimens collected about a 100 years ago (Délye *et al*., 2013a). Under herbicide selection, strong resistance can develop within a few generations in such populations. This is due to the fact that mutations present as standing genetic variation have raised to certain frequencies and could already more easily establish in the populations (Hermisson and Pennings, 2005). This is further facilitated by high census population sizes, which can rapidly emerge in years with insufficient weed control and therefore provide a large genetic resource for resistance mutations (Menchari *et al*., 2007).

Besides standing genetic variation, another potential source for TSR mutations in these organic fields could be gene flow and seed dispersal by wind, pollen flow, agricultural machinery or wildlife (Colbach and Sache, 2001; Somerville *et al*., 2019). However, in the case of the organically farmed fields, we find not only the TSR haplotypes but also a corresponding wild‐type haplotype, which differs by only one mutation in the entire 13.2 kb amplicon. This is true in all three cases, making it likely that the TSR mutations arose from these wild‐type alleles independently in the fields – noticeably, for each field, there are also three pairs of wild‐type haplotypes that differ by only a single mutation from each other. Moreover, we find the wild‐type haplotypes in two out of the three cases with higher frequency than the corresponding TSR haplotypes (Figure 4), further suggesting that gene flow as a source is not very likely. Instead, the abundant plants with the matching wild‐type haplotypes in these fields are the most likely source for different TSR mutations of independent origin.

### TSRs will likely remain in fields for many decades even without selection

Adaptation to a new environment is often constrained due to pleiotropic fitness effects in previous conditions (reviewed in Purrington (2000)). TSR mutations in weed populations represent this special case in agricultural fields, where they become highly beneficial under herbicide application and rise in frequency. However, in the absence of herbicide selection, they have predominantly neutral or even detrimental effects (Du *et al*., 2019; Menchari *et al*., 2008; Tardif *et al*., 2006; Vila‐Aiub *et al*., 2015). At least one *ACCase* mutation, Ile1781Leu, is known to be beneficial under neutral conditions (Délye *et al*., 2013b; Wang *et al*., 2010). On the other hand, there have been reports of fitness effects in several TSR mutations in the absence of selection, although quite often the differences do not persist when assessed in realistic field conditions or in competition with other plants (Du *et al*., 2019). Unfortunately, the fitness proxies used – for example, biomass, netto assimilation rate, relative growth rate, plant height, leaf area ratio, seed production, or *ACCase*‐specific activity – are difficult to compare and it is difficult to translate these observations into uniform estimates of selection coefficients (Anthimidou *et al*., 2020; Sabet Zangeneh *et al*., 2016; Vila‐Aiub *et al*., 2009, 2015, Yu *et al*., 2007).

**Figure 5:**
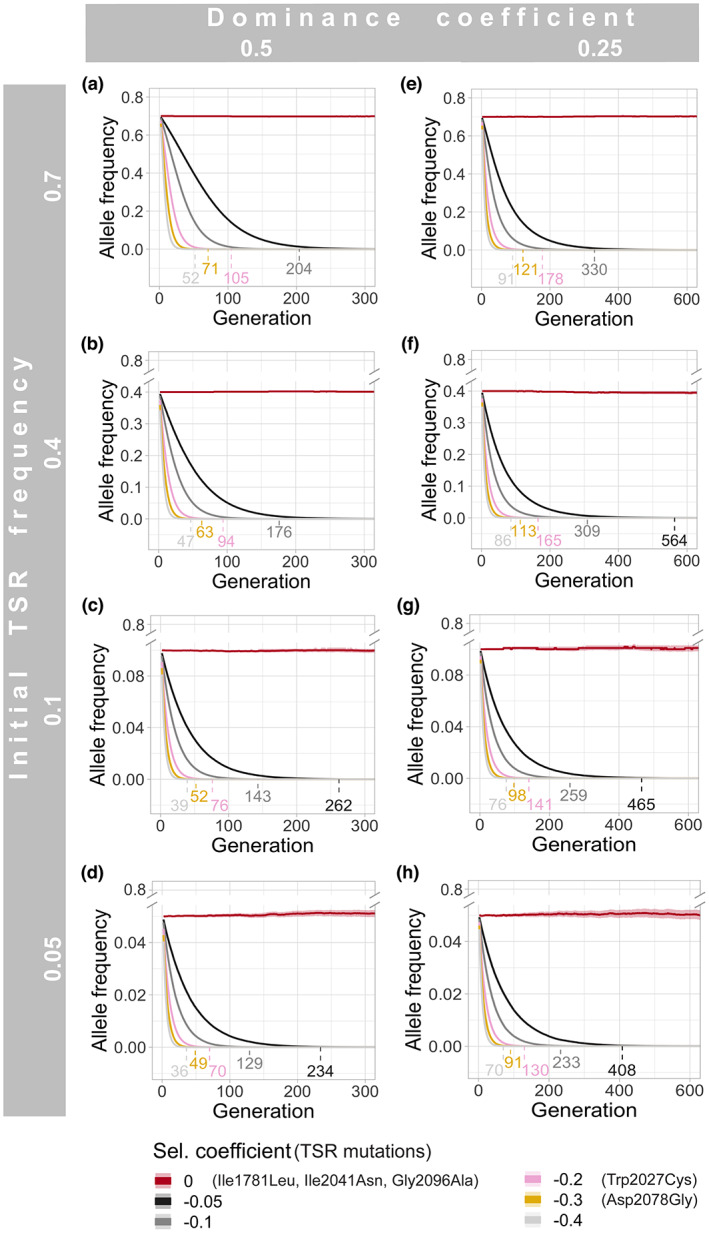
Simulations of the number of generations in which TSR alleles remain in *A. myosuroides* field populations in the absence of selection, assuming different selection coefficients, as estimated from fitness experiments (Du *et al*., 2019; Menchari *et al*., 2008). While homozygous individuals suffer the full consequences of deleterious TSR mutations, we simulated two different dominance coefficients for heterozygous allele states: an intermediate codominance of 0.5 (a–d) and a more recessive coefficient of 0.25 (d–h). The coloured numbers above the x‐axis indicate the average number of generations at which the mutations shown at the bottom are lost in the different scenarios. Means and 0.95 confidence intervals per parameter combination are shown.

Because herbicide resistance has become such a serious problem in recent decades, it is important to learn whether the foregoing herbicide application for certain intervals is sufficient to remove a given TSR mutation from a field population via genetic drift. To tackle this question, we generated forward‐in‐time simulations with the software SLiM (Haller and Messer, 2019). While most studies focus on a few individuals of many populations (Délye *et al*., 2004c; Menchari *et al*., 2006), the depth of our pools allows us to assess more realistic haplotype frequencies of TSRs from our empirical data set (Figure 3). We used high (0.7; Figure 5a,e), intermediate (0.4; Figure 5b,f) and low (0.1 and 0.05; Figure 5c,d,g,h) initial TSR frequencies for our simulations, considering that many TSRs are usually present in the heterozygous state. The simulated selection coefficients ranged from 0 (no detrimental effect in the absence of herbicide selection) to 0.4 (40% fitness cost in the absence of herbicide selection). Within this range, we included the reported selection coefficient estimates for TSR mutations Trp2027Cys and Asp2078Gly, for which under realistic field scenarios, seed production was significantly reduced by 20% and 30%, respectively (Du *et al*., 2019). Other parameters, such as effective population size, mutation and recombination rate, were obtained from the literature (Bauer *et al*., 2013; Kersten *et al*., 2023; Yang *et al*., 2017). For each mutation and initial allele frequency, we simulated two different dominance coefficients derived from fitness experiments in *A. myosuroides* (Menchari *et al*., 2008), an intermediate, codominant coefficient of 0.5 (Figure 5a–d), and a recessive coefficient of 0.25 (Figure 5e–h). We conducted 400 independent SLiM simulation runs per parameter combination and estimated the average number of generations for a TSR to be removed from the population by genetic drift.

The SLiM simulations indicated that under the best‐case scenario, with a low initial allele frequency (0.05), a strong deleterious selection coefficient (−0.4), and codominance (0.5), it would take, on average, 36 generations (the average number of generations at which all simulations reach 0 allele frequency) until the TSR mutation is lost (Figure 5d). Unfortunately, farmers often recognize a resistance problem in their fields only once the TSR mutations have already risen to high frequency. Furthermore, most TSR mutations do not seem to reach such a strong deleterious fitness effect (Du *et al*., 2019; Menchari *et al*., 2008). For example, under a milder selection coefficient of −0.1 (still below what has been reported for Trp2027Cys; Du *et al*., 2019), codominant mutations would persist in a non‐treated field, on average, for up to 204 generations (when the initial allele frequency = 0.7), and up to 330 generations when more recessive. Since these numbers of generations are mostly beyond the lifespan of a farmer, not to mention the economical loss incurred by a field being fallow for decades, additional measures need to be taken to manage and prevent herbicide resistance.

## Discussion

Pool sequencing of amplicons with PacBio HiFi reads is a cost‐effective method for sequencing thousands of samples while preserving haplotype resolution. The *pbaa* clustering software eliminates the need for read alignment against a reference and phasing. Instead, HiFi sequences are clustered directly, preserving the full information contained and reducing reference bias. This opens up new avenues for the discovery of unknown structural variants and genetic diversity. Furthermore, the complete amplicon workflow can be easily established as a high‐throughput method for almost any gene of interest in any organism. A notable exception for any pool‐based approach in a single locus would be the monitoring of recent gene amplification, such as the alternative resistance mechanism in response to glyphosate discovered for the *EPSPS* gene (Gaines *et al*., 2010).

Importantly, in cases, where the genes of interest are shorter than *ACCase*, long amplicons that include intergenic sequence up‐ and downstream of the gene are likely to provide even higher resolution of alleles, as variation in intergenic sequences is usually higher than in more constrained genic regions. Although *pbaa* was able to resolve haplotypes that differ by a single variant since the technology has difficulties with homopolymers, it would be prudent to mask these, at least beyond a certain length (e.g. >6 bp).

A valid concern is whether PacBio HiFi technology is appropriate for applications that require the fast return of sequencing data. In a commercial setting, a large number of samples collected in a short‐time frame will help to quickly fill an entire SMRT cell. Alternatively, one could use another long‐read technology, such as Oxford Nanopore Technologies. Although single‐read accuracy has been limiting for Oxford Nanopore data, the latest developments with duplex read promise to reach q30 (https://github.com/nanoporetech/duplex‐tools), as used here for PacBio HiFi‐based amplicons.

Based on the two population studies we conducted in *A. myosuroides*, Europe‐wide and in Germany, we can conclude that herbicide resistance arises independently in different field populations. This puts farmers and consultants in charge to investigate their fields carefully and obtain the status quo of their fields in terms of the resistance situation because once a resistance mechanism is established in a field population, it is highly unlikely to be lost over the course of a human lifetime, even after herbicide application is stopped. The variation in resistance across the fields sampled in the current study supports the assertion that weed management strategies should focus on the field level, requiring accurate and up‐to‐date information on the prevalence of herbicide resistance in a given field. A recent survey in Germany found that while only 20% of agricultural fields suffered from high levels of infestation with *A. myosuroides*, resistance to the *ACCase* inhibitor pinoxaden could be detected in 80% of samples (Hess *et al*., 2022). This indicates that successful resistance management requires precautionary control of the census population size of the weed. Management strategies should therefore focus not only on chemical but also non‐chemical measures, such as delayed seeding, moldboard ploughing and crop rotation (Lutman *et al*., 2013; Moss *et al*., 2007).

## Experimental procedures

### European sample collection

The European collection was provided by BASF. Amplicon sequencing data of 22–24 single individuals from 47 populations has been described (Kersten *et al*., 2023). For this study, we selected nine of those populations containing TSR mutations and resowed and sequenced pools of 200 individuals to assess the potential of *pbaa* clustering in pools versus individuals.

### German sample collection and phenotyping

In the course of a Germany‐wide herbicide resistance assessment (2019), a collection of *A. myosuroides* seeds on 1369 agricultural fields was conducted (Hess *et al*., 2022) All samples came from fields sown with winter wheat or triticale in the year of sampling and were screened in a biotest prior to sequencing. Seeds were sown in sandy‐loam substrate and treated at BBCH 12/13. Two *ACCase* inhibitors were used for the screening, Axial*®* (50 g/L of pinoxaden and 12.5 g/L cloquintocet‐mexyl) and Focus*®* Ultra (100 g/L cycloxydim). Herbicide application was done with 200 L water in a Research Track Sprayer Generation III using a Teejet‐8002‐EVS‐Nozzle and field rates of 1.2 L/ha for Axial*®* and 2.5 L/ha for Focus*®* Ultra. A visual assessment of the efficacy was done 21 days after treatment. All plants were screened in two replicates together with well‐characterized standard populations. Sixty‐four samples were later chosen based on the number of seeds available to conduct further tests, the suitability to form regional clusters, and variations in the degree of efficacy of the tested herbicides. Besides two samples from organic farms, all other samples were collected from conventional farms.

### Growth conditions, harvesting and DNA extraction

All seeds were sown in a standard substrate (Pikiererde Typ CL P, Cat.No EN12580; Einheitserde) and stratified at 4 °C in a climate chamber. Then they were transferred to the greenhouse at 22 °C with 16 h daylight. For the pilot experiment, we harvested 200 individuals per pool in the European collection. For the German data set, 150 individuals were collected from each population. To ensure equal representation of all individuals per pool, grass leaves were cut using a 2.5 cm size template (ca. 10 mg leaf material per plant). Care was also taken to ensure that the leaves were of similar width. All pool samples were collected in 50 mL Falcon tubes filled with 4–5 metal beads and ground with a FastPrep‐24™ 5G tissue disruptor using the CoolBigPrep™ 2 × 50 mL‐Adapter filled with dry ice (Prod. No machine: 15260488, Prod. No adapter: 11471525; MP Biomedicals, Irvine, CA).

DNA purification was performed as detailed in our online hands‐on DNA extraction protocol in GitHub. Briefly, 300 mg of plant leaf powder per pool was incubated for 60 min at 60 °C in 800 μL of lysis buffer (100 mm Tris pH 8, 50 mm EDTA pH 8, 500 mm NaCl, 1.3% SDS and 0.01 mg/mL RNase A) in a 2 mL screw cap tube. After centrifugation at 12 000 *g* for 1 min, 200 μL of the supernatant were transferred to a fresh 2 mL Eppendorf Safe‐Lock tube (Prod. No. 0030120094). To precipitate proteins, 65 μL of 5 m potassium acetate was added to each sample. After vigorous vortexing and a short spin, samples were incubated for 15 min at −20 °C. Following a centrifugation step at 12 000 *g* for 1 min, 200 μL of supernatant was transferred to a fresh 2 mL Eppendorf tube. A first cleanup was performed for 5 min by adding 300 μL of 0.4% solution of SeraMag™ SpeedBead Carboxylate‐Modified [E3] Magnetic Particles (Prod. No. 65152105050450; GE Healthcare). After placing the tube on a magnet, the supernatant was discarded and beads were washed twice with 80% ethanol while keeping the tube on the magnet. Elution was performed with 50 μL of water. A second cleanup was performed with 50 μL of 0.4% solution of SeraMag™ beads, and ethanol washes and elution were done as before.

### *ACCase* amplicon generation and PacBio sequencing

To generate the *ACCase* amplicons, we used a direct dual barcoding approach with target‐specific primers (24 forward and 16 reverse) that had the barcode sequences in their 5′ ends (Data S1). We conducted four independent PCR reactions (using different primer pairs) for each pool. For each PCR, we use 50 ng of DNA as a template. The number of template copies in 50 ng of input DNA of a genome estimated to be 3.56 Gbp (Kersten *et al*., 2023) was estimated to be 13012 according to the following equation:

$$\;\text{Number of copies}=\frac{\text{Amount input}\;\mathrm{DNA}\;\left(\mathrm{ng}\right)\times 6.022\times 10^{23}\;\left(\text{molecules}/\text{mole}\right)\;}{\text{Length of haploid genome}\;\left(\mathrm{bp}\right)\times 1\times 10^{9}\;\left(\mathrm{ng}/g\right)\times 650\;\left(g/\text{mole of}\;\mathrm{bp}\right)}$$

That is, 32.5 and 43.4 copies per haploid genome per PCR reaction for pools of 400 and 300 diploid individuals, respectively.

The 13.2‐kb‐long target region was amplified using a master mix reaction with 1 μL Forward indexing primer (5 μm), 1 μL Reverse indexing primer (5 μm), 4 μL 5× Prime STAR buffer, 1.6 μL dNTPs (2.5 mm each), 0.4 μL Prime STAR GXL polymerase (1.25 U/μL) (R050B; Takara Bio Inc., Shiga, Japan), filled up to 20 μL with water in a two‐step PCR reaction with 28 cycles (denaturation: 98 °C, 10 s; annealing: 68 °C, 11 min; final extension: 72 °C, 10 min; hold: 4 °C). For a quality check, 5 μL of each amplicon pool was visualized on a 0.8% agarose gel, and the concentration was determined with a Qubit™ system. Then, all amplicons were pooled equally into a large pool, bead‐cleaned and size‐selected using a BluePippin system (Sage Science, Beverly, MA) with High‐Pass Plus 0.75% agarose cassettes, 15 kb (342BPLUS03; Biozym Scientific GmbH, Germany). Only fragments larger than 10 kb were retained (Figure S1). The correct fragment size selection was verified with a Femto Pulse system (Agilent, Santa Clara, CA). The PacBio library was created following protocol no. 101‐791‐800 version 01 (June 2019) with the SMRTbell Express Template Prep Kit 2.0 (part number 100‐938‐900). Sequel® II loading was performed according to manufacturer specifications with Sequel® II Binding Kit 2.0 and Int Ctrl 1.0 (part number 101‐842‐900). A detailed hands‐on amplicon protocol can be found in Github.

### Generation and demultiplexing of q30 HiFi reads

Pre‐processing steps were carried out with PacBio tools (https://github.com/PacificBiosciences/pbbioconda). This included the generation of circular consensus sequences (ccs) with ccs v6.0.0 with a minimum predicted accuracy of 0.999 (q30), demultiplexing of pools with lima v1.11.0 with parameter settings ‘‐‐ccs ‐‐different ‐‐peek‐guess ‐‐guess 80 ‐‐split‐bam‐named ‐‐min‐ref‐span 0.875 ‐‐min‐scoring‐regions 2’, and conversion of the resulting bam to fastq format with bam2fastq v1.3.0.

### *Pbaa* clustering

Prior to the *pbaa* clustering, we concatenated the q30 HiFi reads of all PCR reactions corresponding to each pool and normalized the number of reads in each pool to the population with the lowest read counts of each data set by random sampling with fastqtools v0.8.3 (fastq‐sample ‐n<read_number>) (https://github.com/dcjones/fastq‐tools) and indexed each pool with samtools faidx v1.9 (Li *et al*., 2009). In the European collection, we used 16 000 reads per pool and in the German collection 5300 reads. Furthermore, one population in the German data set did not have enough reads; therefore, it was excluded from further analyses.

The provided guide sequence for the reference‐aided clustering approach covered the complete *ACCase* gene sequence and originated from a sensitive plant of a northern Germany reference population (WHBM72 greenhouse standard APR/HA from Sep. 2014) (sequence provided in the GitHub repository for this project).

In the European data set, *pbaa* v1.0.0 (commit 691333c) clustering was performed with ‐‐min‐read‐qv 30 ‐‐max‐alignments‐per‐read 16 000 ‐‐max‐reads‐per‐guide 16 000 ‐‐pile‐size 50 ‐‐min‐var‐frequency 0.4 ‐‐min‐cluster‐read‐count 20 ‐‐min‐cluster‐frequency 0.00125. In the German data set, we used the following adjusted parameters: ‐‐max‐alignments‐per‐read 5300 ‐‐max‐reads‐per‐guide 5300 ‐‐pile‐size 25 ‐‐min‐var‐frequency 0.4 ‐‐min‐cluster‐read‐count 9 ‐‐min‐cluster‐frequency 0.0017 (https://github.com/PacificBiosciences/pbAA). Finally, to extract the consensus sequences generated in the clustering step, including meta information of each haplotype, and re‐orient them – when necessary – in the forward orientation, we used a homemade script, which can be found in the dedicated GitHub for this study.

### *Pbaa* validation in the European data set

All clusters inferred by *pbaa* in the pools and all unique haplotypes from the individuals were combined into a joint fasta file per population. MAFFT v7.407 was used for the multiple alignments (−‐thread 20 ‐‐threadtb 10 ‐‐threadit 10 ‐‐reorder ‐‐maxiterate 1000 ‐‐retree 1 ‐‐genafpair) (Katoh and Standley, 2013) and PGDSpider v2.1.1.5 to transfer the multiple alignment fasta file into a nexus formatted file (Lischer and Excoffier, 2012). The maximum‐likelihood (ML) tree was generated with RAXML‐NG v0.9.0 using the GTR + G model and 1000 bootstraps (Kozlov *et al*., 2019). The minimum spanning network was inferred and visualized with POPART v.1.7 (Leigh and Bryant, 2015). The TSR information for the colouring of the haplotype tree and network was retrieved from a classical alignment of the *pbaa* clusters to the *ACCase* reference gene (see Section ‘Annotation of TSR mutations’). The resulting VCF was loaded and manipulated in R to annotate the ML tree and minimum spanning network. Used R packages can be found in Table S1.

Based on the multiple alignments per population, haplotypes in the pool and individual datasets were counted with the R package ‘haplotypes’ (https://cran.r‐project.org/web/packages/haplotypes/haplotypes.pdf) and summarized in Table 1. Haplotype frequencies were calculated with homemade R scripts and the correlations of individual and pool haplotype frequencies were calculated and visualized using the packages ‘ggpubr’ (https://github.com/kassambara/ggpubr/) and ‘ggplot2’ (Wickham, 2016).

### Comparison of conventional SNP mapping with *pbaa* clustering in the German data set

For the conventional alignment and SNP calling, the reads of each pool were aligned to the *ACCase* reference sequence with pbmm2 (https://github.com/PacificBiosciences/pbmm2). All resulting bam files were merged, sorted and indexed with samtools v1.9 (Li *et al*., 2009). SNP calling was performed with freebayes v1.3.2 (freebayes ‐f $REF ‐‐min‐mapping‐quality 20 ‐‐min‐alternate‐fraction 0.005 ‐‐pooled‐continuous ‐‐report‐monomorphic) (Garrison and Marth, 2012). All single VCF files were compressed, indexed and merged using tabix v0.2.5 (Li, 2011). Before extracting allele depth (AD) and total depth (DP) information for SNPs at TSR positions to compare REF and ALT counts, the multi‐allelic positions were split into multiple rows of biallelic calls with bcftools v1.9‐15‐g7afcbc9 (bcftools norm ‐m ‐any ‐Oz) (Danecek and McCarthy, 2017), followed by converting the variants into a table using the VariantsToTable function from GATK 4.1.3.0 (Van der Auwera *et al*., 2013). The table was loaded and manipulated in R version 3.6.1 (Team, 2018), and allele frequencies were plotted with the R package ‘ggplot2’ (Wickham, 2016).

### Annotation of TSR mutations

To annotate the clusters generated with the *pbaa* clustering approach with the TSR information, the single cluster fasta files were transferred to *fastq* files in which all bases were assigned quality ‘I’, with Fasta_to_fastq (https://github.com/ekg/fasta‐to‐fastq). Afterwards, the *fastq* files containing a single read representing the corresponding cluster were aligned to the *ACCase* reference with minimap2 v2.15‐r913‐dirty (Li, 2018). The resulting bam file was sorted and indexed with samtools v1.9 (Li *et al*., 2009) and the read groups were adjusted with Picard's v2.2.1 function AddOrReplaceReadGroups (RGID = $SAMPLE RGLB = ccs RGPL = pacbio RGPU = unit1 RGSM = $SAMPLE) (http://broadinstitute.github.io/picard). Variant calling, which results in the VCF file, was performed with the HaplotypeCaller from GATK 4.1.3.0 (‐‐R $REF ‐‐min‐pruning 0 ‐ERC GVCF), followed by GenotypeGVCFs with standard settings (Van der Auwera *et al*., 2013). Variants in the resulting VCF file were annotated with SnpEff v4.3t (Cingolani *et al*., 2012).

### Organic fields with TSR mutations of independent origin

The TSR information of the organic fields was extracted from the previously described haplotype clustering in the German data set. The clusters in the BAM files were coloured with *pbaa* v.1.0.0 bampaint and visualized in the Integrative Genomics Viewer IGV_2.11.9 (Robinson *et al*., 2011).

### SliM simulations

We performed forward simulations with SLiM v3.4 (Haller and Messer, 2019) under Wright‐Fisher model assumptions to determine the number of generations that TSR mutations persist in agricultural fields without being under herbicide selection. A population size of 42 000 individuals was assumed, following calculations from the previous publication (Kersten *et al*., 2023). Similarly, we adopted the mutation rate of 3.0 × 10^−8^ (Yang *et al*., 2017) and genome‐wide average recombination rate of 7.4 × 10^−9^ (Bauer *et al*., 2013) from maize, a diploid grass with a comparable genome size. We modelled a range of selection coefficients (s_i_) from 0 to −0.4, covering values based on literature that compared seed production of wild‐type and mutant American sloughgrass (*Beckmannia syzigachne* Steud.) plants during competition with wheat plants under field conditions (*s*_*i*_ = −0.2 for Trp2027Cys, and *s*_*i*_ = −0.3 for Asp2078Gly) (Du *et al*., 2019). We used two dominance coefficients (*h*_*i*_) 0.5 and 0.25 for the TSR mutations as reported in *A. myosuroides* (Menchari *et al*., 2008). The fitness model for individuals carrying a homozygous TSR mutation is 1 + *s*_*i*_, and for a heterozygous one is 1 + *h*_*i*_ * *s*_*i*_. Initial haplotype frequencies were extracted from our empirical pool data and set to 0.05, 0.1, 0.4 and 0.7. We performed 400 independent SLiM runs per parameter combination and calculated the mean values and the 0.95 confidence intervals in R with the package ‘rcompanion’ (https://rcompanion.org/handbook/). Visualization was done with ‘ggplot2’ (Wickham, 2016).

## Funding

S.K. was supported by a stipend from the Landesgraduiertenförderung (LGFG) of the State of Baden‐Württemberg. F.A.R. was supported by a Human Frontiers Science Program (HFSP) Long‐Term Fellowship (LT000819/2018‐L). The majority of funding was provided by the Max Planck Society.

## Conflict of interest

J.H. is the founder and M.H. is the owner of Agris42, a company providing herbicide resistance testing services and weed management consultation to farmers. D.W. holds equity and S.K. is an employee of Computomics, which advises breeders. Z.N.K. is an employee and shareholder of Pacific Biosciences, a company developing single molecule‐sequencing technologies. Other authors declare no competing or financial interest.

## Author contributions

Conceptualization: F.A.R.; Investigation: S.K. with support from F.A.R., J.H. and M.H.; Software: Z.N.K.; Formal Analysis: S.K.; Resources: J.H. and M.H.; Writing – Original Draft: S.K.; Writing – Review and Editing Preparation: S.K., F.A.R. and D.W.; Visualization: S.K.; Supervision: F.A.R., K.S. and D.W.; Funding Acquisition: D.W.

## Supporting information


**Data S1** Phenotyping of German populations, target‐specific primers with barcode sequences attached, and barcodes to sample correspondence.

**Figure S1** Insert size distribution of the PacBio amplicon library.
**Figure S2** Correlation between allele frequencies and haplotype frequencies for TSR amino acid positions Trp1999, Ile2041 and Asp2078.
**Figure S3** Correlations between TSR haplotype frequencies and phenotyping with ACCase inhibitors.
**Table S1** R‐packages used for data manipulation and visualization.

## Data Availability

PacBio HiFi q30 reads for each pool have been deposited in the European Nucleotide Archive (ENA; https://www.ebi.ac.uk/ena/browser/home) under project accession number PRJEB53650. Experimental protocols, SLiM simulations and custom scripts to reproduce the analyses in this study are deposited on GitHub (https://doi.org/10.5281/zenodo.7646820).
