# Supplementary material for: Deep haplotype analyses of target‐site resistance locus ACCase in blackgrass enabled by pool‐based amplicon sequencing
Source: Plant Biotechnol J. 2023 Apr 10;21(6):1240–53. doi: 10.1111/pbi.14033 (PMC10214753; doi:10.1111/pbi.14033)
Supplement: Supplementary file 2 — Figure S1 Insert size distribution of the PacBio amplicon library. Figure S2 Correlation between allele frequencies and haplotype frequencies for TSR amino acid positions Trp1999, Ile2041 and Asp2078. Figure S3 Correlations between TSR haplotype frequencies and phenotyping with ACCase inhibitors. Table S1 R‐packages used for data manipulation and visualization. [file PBI-21-1240-s001.pdf]

Supporting Information for

Deep haplotype analyses of target-site resistance locus *ACCase* in blackgrass enabled by pool-based amplicon sequencing

Sonja Kersten[1,2], Fernando A. Rabanal[2,*], Johannes Herrmann[3], Martin Hess[3], Zev N. Kronenberg[4], Karl Schmid[1], Detlef Weigel[2,*]

[1]Institute of Plant Breeding, Seed Science and Population Genetics, University of Hohenheim, Stuttgart, Germany.
[2]Department of Molecular Biology, Max Planck Institute for Biology Tübingen, Tübingen, Germany.
[3]Agris42 GmbH, Stuttgart, Germany.
[4]Pacific Biosciences, Menlo Park, CA, USA.

Authors for correspondence:

Fernando A. Rabanal: fernando.rabanal@tue.mpg.de
Detlef Weigel: weigel@weigelworld.org

This PDF file includes:

Figures S1 to S3
Table S1

Other supporting materials for this manuscript include the following:

Data S1
GitHub repository (https://doi.org/10.5281/zenodo.7646820)

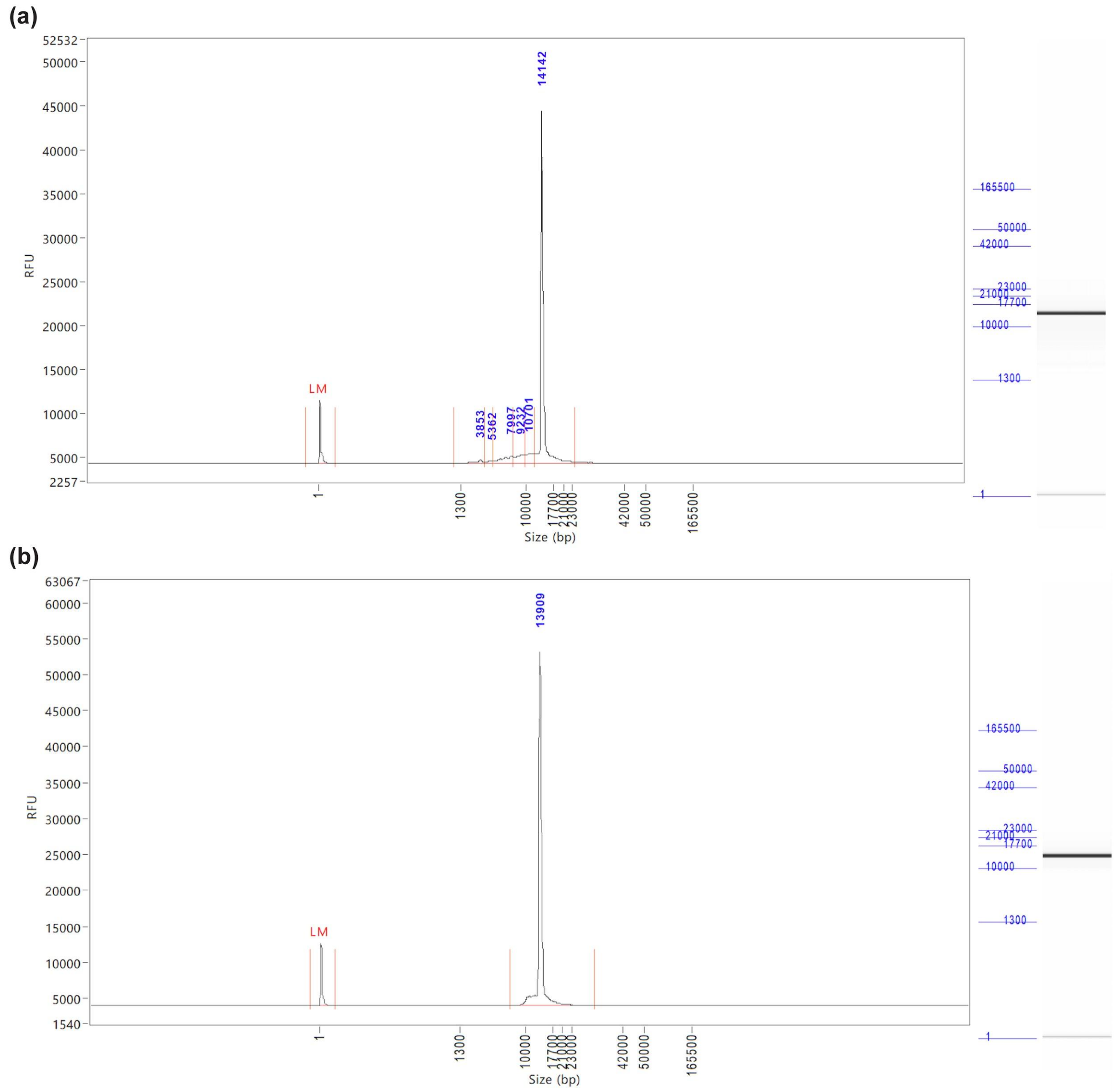

Figure S1. Insert size distribution of the PacBio amplicon library. **(a)** Before and **(b)** after size-selection on the BluePippin instrument as measured on a Femto Pulse System. Only fragments larger than 10 kb were retained.

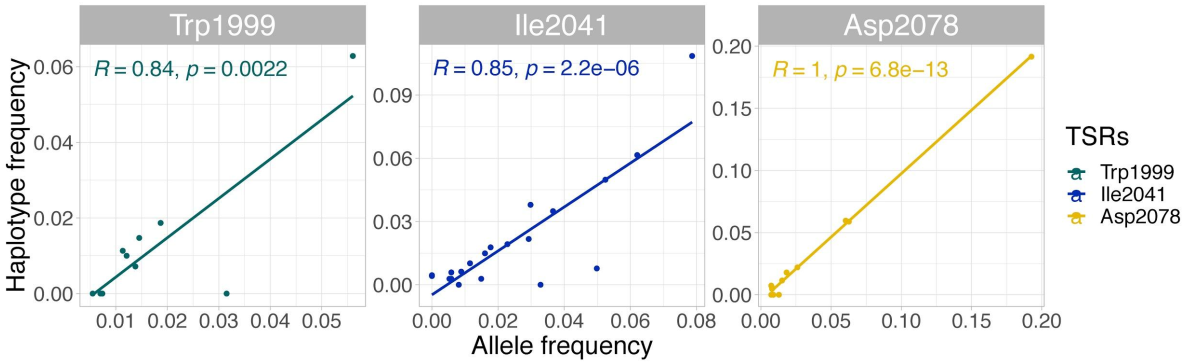

Figure S2. Correlation between allele frequencies and haplotype frequencies for TSR amino acid positions Trp1999, Ile2041 and Asp2078. Correlation coefficients and p-values are shown separately in each TSR panel.

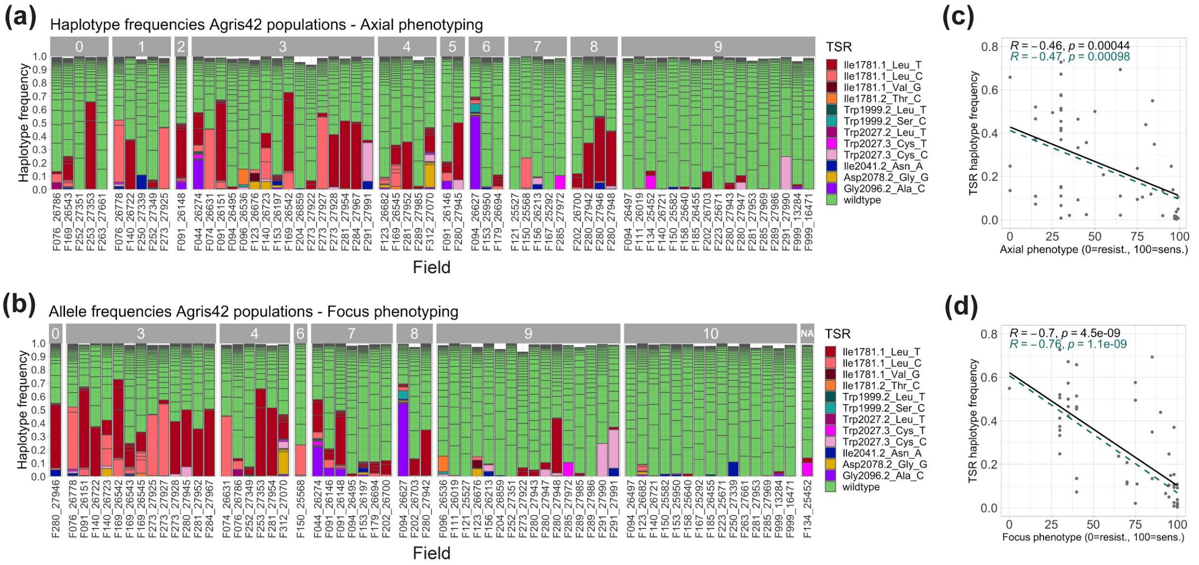

Figure S3. Correlations between TSR haplotype frequencies and phenotyping with ACCase inhibitors. Haplotype frequencies were inferred using *pbaa*. Colors refer to TSR and wild-type haplotypes. **a.** Bins represent the remaining efficiencies of the herbicide Axial® (Bin 0: 0 to 10% efficiency, which means 90–100% survivor plants; Bin 9: 90 to 99% efficiency). Correlation coefficients and p-values for TSR haplotypes and their respective phenotypes are shown in the panel on the right. **b.** Bins represent remaining efficiencies of the herbicide Focus Ultra (Bin 0: 0 to 10% efficiency; Bin 9: 90 to 99% efficiency; Bin 10: 100% efficiency with 0% survivor plants). **c,d.** Correlation coefficients and p-values for TSR haplotypes and their respective phenotypes are shown in the corresponding panels on the right. Black line shows the correlation of all TSR mutations, the green line only the TSR mutations with reported resistance to the respective herbicides (summarized in Table 3 of Powles and Yu, 2010).

Table S1. R-packages used for data manipulation and visualization.

Package name and version	Reference
dplyr 1.0.2	(Wickham *et al.*, 2020)
ggplot 3.3.2	(Wickham, 2016)
ggpubr 0.4.0	Kassambara, 2020 (ref (https://github.com/kassambara/ggpubr/)
ggtree 1.16.6	(Yu *et al.*, 2017)
haplotypes 1.1.2	Aktas, 2020 (ref https://cran.r-project.org/web/packages/haplotypes/haplotypes.pdf)
plyr 1.8.6	(Wickham, 2011)
rcompanion 2.4.1	Mangiafico, 2016 (https://rcompanion.org/handbook)
reshape 0.8.8	(Wickham, 2007)
tidyr 1.1.2	Wickham, 2020 (ref (https://github.com/tidyverse/tidyr/)
tidyverse 1.3.0	(Wickham *et al.*, 2019)
treeio 1.18.1	(Wang *et al.*, 2020)
vcfR 1.12.0	(Knaus and Grünwald, 2017)
